# Congestion Prediction Modeling for Quality of Service Improvement in Wireless Sensor Networks

**DOI:** 10.3390/s140507857

**Published:** 2014-04-30

**Authors:** Ga-Won Lee, Sung-Young Lee, Eui-Nam Huh

**Affiliations:** Department of Computer Engineering, Kyung Hee University, 1732 Deogyeong-daero, Seochon-dong, Giheung-gu, Yongin-si, Gyeonggi-do 446-701, Korea; E-Mails: gawon@khu.ac.kr (G.-W.L.); sylee@oslab.khu.ac.kr (S.-Y.L.)

**Keywords:** Wireless Sensor Networks (WSNs), congestion prediction, traffic demands analysis, traffic modeling

## Abstract

Information technology (IT) is pushing ahead with drastic reforms of modern life for improvement of human welfare. Objects constitute “Information Networks” through smart, self-regulated information gathering that also recognizes and controls current information states in Wireless Sensor Networks (WSNs). Information observed from sensor networks in real-time is used to increase quality of life (QoL) in various industries and daily life. One of the key challenges of the WSNs is how to achieve lossless data transmission. Although nowadays sensor nodes have enhanced capacities, it is hard to assure lossless and reliable end-to-end data transmission in WSNs due to the unstable wireless links and low hard ware resources to satisfy high quality of service (QoS) requirements. We propose a node and path traffic prediction model to predict and minimize the congestion. This solution includes prediction of packet generation due to network congestion from both periodic and event data generation. Simulation using NS-2 and Matlab is used to demonstrate the effectiveness of the proposed solution.

## 1. Introduction

Modern life has become increasingly convenient with the development and deployment of Information technology (IT) in nearly all areas of life. In particular, the Internet of Things (IoT) is a way to provide services to users using a convergence of both information produced by humans together with that obtained from everyday objects by networking all things and equipment. It is expected that this will play a part in a new service ecosystem capable of judging situations independently, performing tasks without human intervention, and providing information collected through the interactions between many different objects.

Ubiquitous technology, which has been identified as a key element of the IoT, has developed rapidly based on Wireless Sensor Networks (WSNs) [1]. IoT is well known as a new communication system for the physical world and Internet connection via WSNs [2]. Accordingly, there is a trend to move from individual small WSNs to multiple large-scale networks in which multiple WSNs are integrated and share information. The role of WSNs has changed from simple surveillance and gathering of information to more critical tasks. As a result, plans for how to process and share large amounts of sensor data and maintain quality of service (QoS) guarantees are being developed [3]. There are several projects underway in the United States, Europe, Japan, and other locations to realize ubiquitous computing based on the IoT, in which all objects are networked and can exchange information [4]. In Korea, a wide range of projects and demonstrations have been initiated under the leadership of the government.

There is a lot of research underway to solve a variety of related obstacles to implement the IoT, such as methods for autonomic networking configuration among tens of thousands of nodes, what fusion method should be used for a large-scale heterogeneous network, what exchange method is most efficient for dynamic autonomic data, methods for processing of uncertain data without affecting the reliability of network information, and other service-related issues such as software theoretical modeling to provide comprehensive intelligence services to users and Service Delivery Schemes [5,6]. However, the most important problem being researched is how to effectively implement the IoT by inter-networking heterogeneous subnets using different infrastructures while considering the cost and quality of service (QoS) of each component and network.

The three most significant components of the IoT are the Internet, mobile networks, and WSNs [7–9]. A wide range of commercial services using existing IP-based networks and mobile networks have been already offered. There are relatively few issues in configuring the IoT, since each company can offer a reasonable quality-adjusted price for basic service while a variety of research projects are ongoing to improve product quality [10,11]. On the other hand, current WSN studies are being conducted on a variety of devices and applications, and commercialization has begun in sectors such as ubiquitous computing for healthcare and smart homes. However, there has been few research reported thus far on totally integrated ubiquitous computing models; thus, there is no management plan, which creates a difficult situation for quality management and coordination. WSN QoS management and improvement are thus urgent problems for IoT configuration [12].

WSNs deliver a data-below topology consisting of a tree structure centered on a sink with wireless communications to the sink. A large Ubiquitous Sensor Networks (USN), consisting of different types of WSNs integrated together, has a polynuclear structure and has a much higher probability of experiencing congestion due to its Multi-Sink, Multi-Event and Mobile Sink characteristics. Communications over wireless networks are generally not reliable compared with those over wired channels [13]. Due to these characteristics, there is a high probability of congestion in the network around the sinks. In addition, effective modeling is required due to the specific characteristics of WSNs, such as wireless communication between the sensor nodes and their constraints as small, low-power devices. Furthermore, common QoS requirements should be improved since WSNs have different QoS requirements for each application. Along with common QoS requirements, there is also a need for higher network throughput to minimize message delay, a need to improve data transmission reliability, and a need for network longevity. In order to satisfy these requirements, congestion and unnecessary traffic should be minimized and techniques to improve the reliability of data transmissions are required.

**Figure 1.**
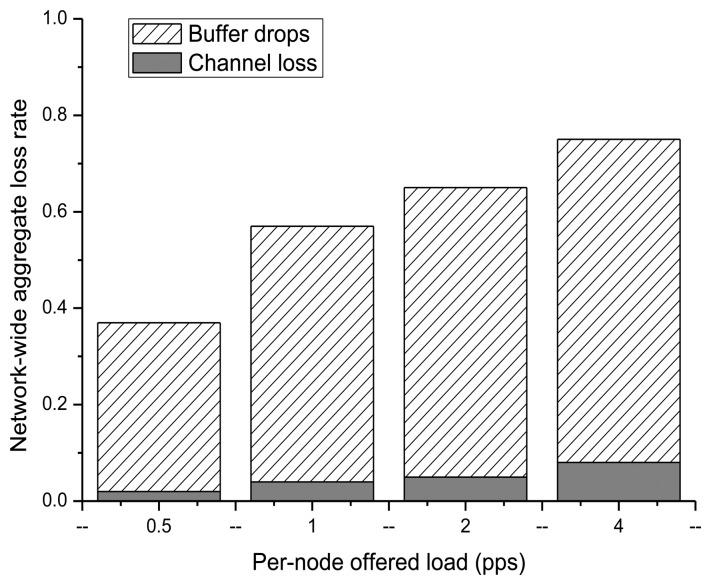
Wireless Sensor Networks (WSN) Quality-Congestion dramatically degrades channel quality [14].

Figure 1 illustrates that most transmission failures caused by congestion during data transmission in WSNs occur because of buffer drop and channel loss [14]. From this, it can be seen that network throughput decreases in the presence of congestion in the WSN, thus increasing the message delay. Therefore, in order to satisfy the common QoS requirements, study of congestion management, improvement of transmission reliability, and node energy management are required.

Previous studies addressing data transfer or QoS guarantees have been divided into several categories: (1) techniques to reduce the amount of data generated by the sensor nodes [14,15]; (2) techniques to detect and control congestion depending on the status of the sensor nodes' queues [16,17]; (3) techniques to detect congestion using packet service times [18]; (4) techniques to adjust the throughput at the receiving node by passing the congestion state on to neighboring nodes [19]; and (5) techniques using packet priority [20]. These techniques all have the disadvantage of requiring an extra transmission to send a congestion signal. In addition, when congestion is detected, the throughput is reduced by dropping the packet and adjusting the sampling period of the node at which congestion occurred.

The purpose of this study is to maximize the utility of the network by predicting the occurrence of congestion through WSN modeling. In this paper, in order to detect congestion in sensor networks, generated periodic and non-periodic packets are predicted by node depending on the placement of the sensor nodes themselves. The prediction reliability can be improved by predicting the transmission time based on the network types.

This study is organized as follows: In Section 2, related techniques are examined. In Section 3, the proposed WSN modeling techniques are explained. Section 4 describes an experiment measuring the accuracy of the proposed method. Finally, Section 5 presents the conclusions of this research.

## 2. Related Works

### 2.1. Congestion Control Methods

When congestion occurs in a sensor network, buffer overflows and packet collisions occur because the competition for wireless communications resources can cause dropped packets. Such data losses result in duplication due to retransmission, which is energy inefficient. Therefore, congestion detection and control techniques matched to the characteristics of sensor networks are required. Various congestion control schemes to solve these problems have been studied, and are detailed in Table 1.

If a certain amount of data is required for a specific area, Event-to-Sink Reliable Transport (ESRT) [15] is a technique that can adjust the reliability of a specific event occurring based on that event in the entire field. The optimal operating area can be determined by calculating the amount of incoming data from the data requirements of the sampling period and sink. However, it is applicable only in terms of the reliability of the entire field, and not the reliability of each event; thus, there is a lack of fairness for most events. In addition, the sink broadcasts packets for throughput adjustment in order to control the congestion, which has the disadvantage of increased energy consumption.

Congestion Detection and Avoidance (CODA) [16] is a technique to avoid conflicts by sending a congestion flag when the queue is beyond the limits of the parent node. CODA can detect and control congestion by calculating the channel loading and the arrival time of the packet through node buffers, as well as by channel monitoring. When congestion occurs, the throughput can be reduced significantly by use of the Additive Increase/Multiplicative Decrease (AIMD) technique or by packet dropping. However, a way is needed to compensate for loss of data due to channel congestion and errors due to the Bit Error Rate (BER). However, even though CODA can be controlled, there is no technique available for ensuring data reliability or identifying the state of channel loading that causes high energy consumption.

In Adaptive Compression-based congestion control Technique (ACT) [17], congestion is detected using queue monitoring with a multi-queue threshold. When congestion occurs, adaptive flow control techniques and compression techniques are used to adjust the packet transmission interval. In the flow control technique, data loss is prevented and data throughput is adjusted with compression to improve fairness as sensor nodes around the sink prevent queue overflow. However, a congestion bit was added in the general data packet header to eliminate the need for additional traffic packets for congestion control. Congestion status updates are also not possible when there is a general loss of data, and congestion notification across the whole network is difficult when configuring a wide range of sensor networks.

The congestion control and fairness (CCF) [18] technique detects the service time and congestion using the packet service information, and adjusts congestion by controlling throughput depending on the number of child nodes. If the packet error rate is high or the amount of transfer data per node is different, the efficiency can be lowered.

SenTCP [19] is an open-loop and hop-by-hop congestion control technique for regulating the transmission rate of the receiving node by continuously passing congestion status information to neighbor nodes in order to allow them to reduce network congestion. SenTCP calculates the congestion coefficient according to buffer status information, which is passed to the neighboring nodes. The node receiving the congestion information may then transmit to the other node depending on the information. Unlike CODA, SenTCP does not include techniques to ensure the reliability of the data, and has the disadvantage that the throughput control algorithm determines performance according to seven parameters.

The priority-based congestion control protocol (PCCP) [20] gives priority on a node-by-node basis, and in the event of congestion, it transmits packets according to priority. If a packet cannot be sent to the parent node because of congestion, it will attempt to find a new parent node through multiple paths.

### 2.2. Wireless Sensor Network (WSN) Traffic Modeling Method

The traffic model in wired and wireless network has been investigated. We can divide the traffic prediction model into two categories: traditional network and WSN. Traditional network traffic prediction models are Markov model, Poisson model, linear regression model and time series forecasting model [21]. Customary, Constant Bit Rate (CBR) traffic model and Markovian model is generally used in WSNs without any discussion as to whether this is appropriate of not [22]. Otherwise, the traffic model in WSNs has not been investigated much. Table 2 shows the previous traffic-related work in WSNs.

To defend attacks in WSNs, Intrusion Detection [23], Auto-Regressive model (AR) [24], Auto-Regressive Moving Average model (ARMA) [25], Swam intelligence Auto Regressive Moving Average model (S-ARMA) [21], and Anomaly Detection [26] were proposed. Other WSN traffic prediction algorithms include the Poison model [27], Constant Bit Rate (CBR) model [28], and Markov process model [29]. For the traffic analysis and modeling, traffic arrival process, sequence relations among general kinds of packets, and data traffic load distribution were has been introduced. Besides, Mobility-Aware modeling that captures the statistical patterns of the mobility and spatial correlation using mobile agent was presented [22].

WSNs are difficult to predict due to their dynamic nature. Furthermore, traffic dynamics in WSNs are application dependent. Therefore, WSN, which is dynamic and event-driven system, specific traffic prediction model is the prerequisite for the network management [21] such as network optimization, traffic distribution, load balancing, attack detection, *etc*.

## 3. Congestion Prediction Model

### 3.1. Preliminaries

#### 3.1.1. Cobb-Douglas Production

When evaluating the mutual influence of all variables of economics, relative change (*i.e.*, resilience) is typically more useful than absolute value. It has been noted that the value indicating resilience is similar to the derivative of a natural logarithm. Therefore, if an expression can be expressed as a natural logarithm, then resilience analysis will be convenient. The formula that is most convenient for analysis is a linear function of the primary addition, *i.e.*, the equation of the straight line. To satisfy both of these requirements, the Cobb-Douglas production function was defined as follows:

(1) $$Q=AL^{\alpha }K^{\beta }$$

where *Q* is product, *K* is capital, and *L* is labor. *A* is an arbitrary constant with the value of the amount, α and β are constants greater than zero and less than 1, respectively. The Cobb-Douglas production function is a production function of linear homogeneity. If each factor increases its ratio at the same time, the product also grows at the same rate and attribution principles are established.

#### 3.1.2. Transportation Demand Analysis Techniques

Traffic demand analysis is the process of looking at the relevance between traffic demand and socio-economic activities, the analyzing the factors that determine traffic demand and its impact on changes in traffic. Traffic demand analysis is distinguished from traffic prediction, which has a primary goal of producing traffic forecasts for individual links. Whether the results of traffic demand analysis can be used to predict future traffic depends on the ability to understand the impact of factors that affect predictive self-traffic and the ability to predict the various explanatory variables used in transportation demand models. Transportation demand modeling is important for the forecasting process, but there are limits to modeling as a means of prediction. Traffic demand analysis can be used as a model for short-term prediction of traffic volume, but the usefulness of transportation demand models for traffic prediction becomes increasingly limited as the forecast period lengthens [30].

The development of models that can meet the ultimate goal of transportation planning is required. These include a direct demand model for inter-city travel demand analysis, four-step transportation demand forecasting methods for the target area (which is divided into multiple zones for analysis), and a probability choice model to select the best alternative and to maximize the utility among all possible alternatives.

#### 3.1.3. Bureau of Public Roads (BPR) Function

Travel time functions are used to solve traffic problems when planning urban transportation or calculating increases in traffic over time. Existing research on travel time functions can be divided into empirical and theoretical types. The traditional method, used mainly in the 1960s, uses linear equations, exponential equations, logarithmic equations, and Bureau of Public Roads (BPR) for empirical expressions. Campbell and Wordrop models are used for theoretical expressions.

The most widely-used BPR expression was proposed by the United States Bureau of Public Roads in 1964. It shows changes over the passage of time depending on increases in traffic volume. Its performance changes depending on the increase, and it accurately predicts free flow speed and capacity [31].

Conical functions [32] were proposed in order to overcome problems related to the rapid growth of the BPR passage delay functions curve. Other several repair function expression was proposed (Soltman, Overgaard, Traffic Research Corporation, Dafermos, Steenbrink, *etc.*) [33].

### 3.2. Research Objectives and Scope

WSN congestion is caused by the fact that packets produced at each node must be forwarded to a sink [34,35]. In other words, because each node cannot send data directly to the sink, transfers are accomplished through multiple nodes by using a variety of routing protocols. Thus, network congestion occurs as a result of attempting to meet the demand to move these data.

Factors that affect data transfer between the nodes in a WSN are as follows:

- Location of the Source Node
- Location of the Sink Node
- Packet type (Text/Image/Sound Data…)
- Number of transmission paths
- Periodic/Event Data incidence
- Path change in case of the occurrence of Periodic Data/Event Data
- Sink Node change in case of the occurrence of Periodic Data/Event Data
- Transfer method (Protocol)
- Resource of Sensor Node

**Figure 2.**
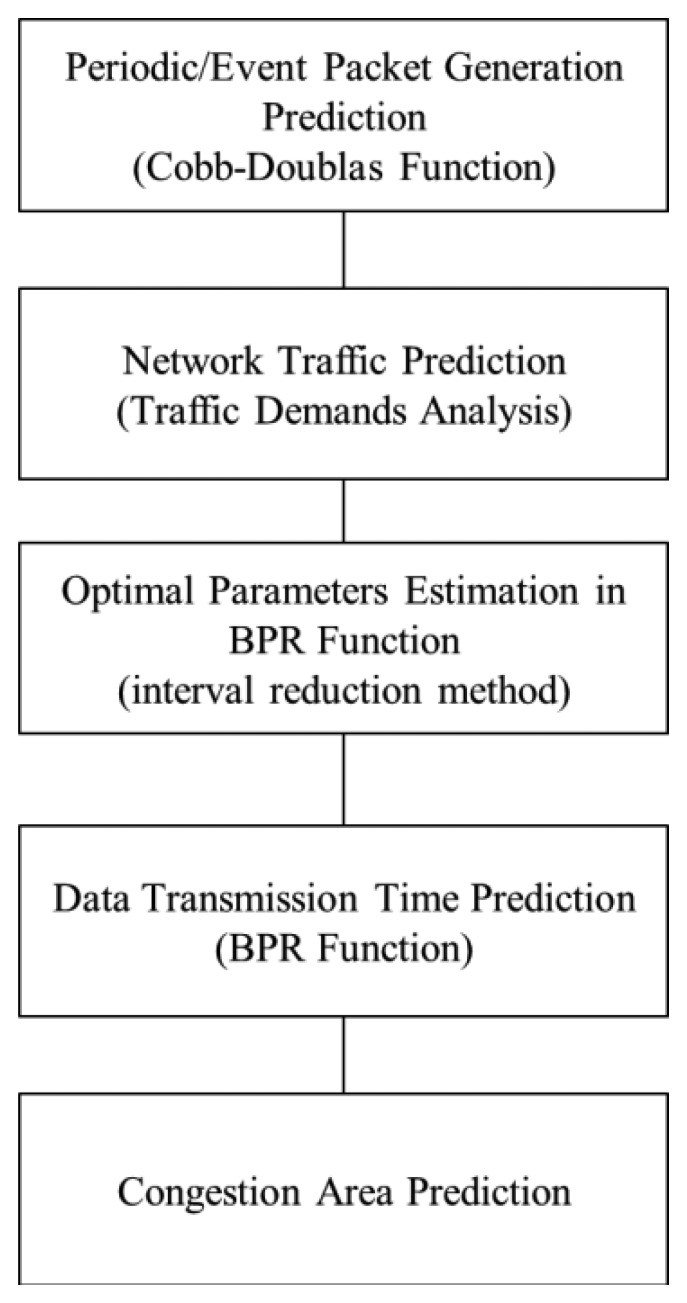
Congestion Prediction Methodology.

The factors that affect data transfer at each node can be represented by a hierarchical system. For example, the type of packet is determined initially according to the sensor node, but the transmission path between the nodes can be determined when network configuration is finished or it can change periodically. In addition, any choice is made based on the cluster unit and district unit, and any other choice is made based on each node. Therefore, the decision can be layered into three steps, as shown in Figure 2:

(1) The entire packet flow can be predicted by dividing it into periodical and event packets and by using the production function from economics after identifying the factors influencing communication between nodes in the WSN.
(2) Traffic can be estimated by using transportation demand analysis techniques.
(3) The congestion zone is predicted by estimating congestion costs through the BPR function after the optimal parameter values are determined according to the network type.

Transmission entails time and node energy consumption; these are both considered to be costs. The throughput that occurs under each different cost is network traffic demand, which is considered to be different from network traffic.

In order to estimate congestion, the number of node-specific packet generations, the average time per node transmission, and the network type must be considered. However, it is difficult to capture all packet information, and it is possible to overestimate the probability when using the average transmission time of the whole network. In addition, if the network was estimated using the arithmetic mean from the average transfer rate without reflecting differences in the type of network, then problems may be underestimated when there is a low-speed network near the sink node and a relatively high-speed network in the outlying areas. The purpose of this study is to identify the factors that determine demand on the WSN network and to predict how these factors impact network traffic.

### 3.3. System Model

The model established in this study is a closed WSN without movement from one sensor network to another, and is assumed to be a mono-centric network with one sink. Packet production is predicted by including periodic data and event data together. In addition, the amount of data being passed over the network can be determined not only by considering end-to-end packet delivery, but also by predicting the number of packets by transmission path. It is assumed that the nodes can move within the WSN.

**Figure 3.**
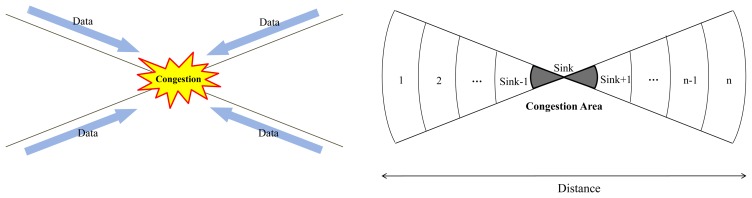
Network Modeling Concept.

The network modeling is shown in Figure 3. The form in which congestion occurs is the basic concept. Periodic and event data are gathered at the sink because commuting and shopping in the city are introduced. The network is divided into N sections in total. The sink is located in the middle of the network, and the N sections are located in the outermost zone [36–39]. The communication radii of each sensor node are all the same, and each node is responsible for all incoming and outgoing data functions. The periodic data and event data are generated in all sections.

### 3.4. Traffic Prediction

#### 3.4.1. Packet Generation Prediction by Node

##### Production Function

This paper uses the concept of production economics to calculate packet production. When evaluating the mutual influence of all variables, relative change, (*i.e.*, elasticity) is very important. Resilience analysis becomes easier when the packet product is expressed as a natural logarithm. The packets produced in the network are modeled using the Cobb-Douglas production function, which models the relationships of the inputs and outputs of the factors of production [40]. The Cobb-Douglas production function is used in economics and is often used as a utility function and is essential to reflect the modeling after analyzing the future effectiveness of the network. If the number of sensing actions and the input power are increased *h* times in the network modeling described in this paper, we can assume that the number of packets is also increased h times, which indicates a constant returns to scale character. The node located in the *i*^th^ area produces packet *D_i_* by using sensing action *S_i_* and Energy *E_i_*. Since the production function was assumed to be a Cobb-Douglas production function, *D_i_* is as follows in Equation (2):

(2) $$D_{i}=A{\left(S^{\mu }\right)}_{i}{\left(E^{\delta }\right)}_{i}$$

Since it is a constant returns to scale function, μ + δ = 1. *A* is a constant with the value of the amount, and represents the number of sensing nodes.

Sensing *S_i_* is determined by the production cost per packet *p_i_* and CPU operations *U_i_*, and energy *E_i_* is assumed to be determined by the production cost per packet *P_i_* and power consumption:

(3) $$S_{i}=\frac{\mu P_{i}}{U_{i}}$$

(4) $$E_{i}=\frac{\delta P_{i}}{r_{i}}$$

This formula was not used for actual traffic prediction in this paper, but was modeled to identify the total production of packets for the total lifetime of the node depending on the node's lifetime and the number of events. There are three node types considered in this study: 100% (node 1), 70% (node 2), and 40% (node 3). When energy is 100%, 100 min of sensing coverage is possible, and when it is assumed that one node is sensed once per minute, the same number of packet nodes from 1, 2 and 3 will be produced in 40 min. However, when comparing the total product per node after 100 min, node 1 produces 1/1 s × 1,000 s = 1,000 packets, node 2 produces 700, and node 3 produces 400. For this type of estimation, network utility analysis may be used for the future by representing the total amount of product and by using the Cobb-Douglas production function according to the amount of energy and sensing. In addition, the derived total number of packets produced can be compared with the traffic prediction of Section 3.4.2.

##### Prediction of the Amount of Data by the Types of Data (Event/Periodic)

Data transmission from the node can be divided into Periodic Data transfers F^w^*_i_*_−sink_ and Event Data transfers F^v^*_i_*_−sink_. Therefore, the Data node from the total throughput is F*_i_*_−sink_ = F^w^*_i_*_−sink_ + F^v^*_i_*_−sink_. The following F*_i_* in Equation (5) is the traffic volume in each traffic zone in Figure 3 showing relay node throughputs:

(5) $$\begin{array}{c} {\text{F}}_{i}=\sum_{\forall k,\;k\neq \text{sink}}{\text{F}}_{k-\text{sink}},\;i=\text{sink}\\ {\text{F}}_{i}=\sum_{\;i\neq \text{sink}}{\text{F}}_{i-\text{sink}}+\sum_{k=1}^{i-1}{\text{F}}_{k-\text{sink}},\;1<i<\text{sink}\\ {\text{F}}_{i}=\sum_{\;i\neq \text{sink}}{\text{F}}_{i-\text{sink}}+\sum_{k=i+1}^{n}{\text{F}}_{k-\text{sink}},\text{sink}<i<n\\ {\text{F}}_{i}={\text{F}}_{i-\text{sink}},i=1,n \end{array}$$

#### 3.4.2. Path Traffic

During WSN data transfer, the decision-making process for network use is as follows:

(1) Traffic Generation: prediction of the number of transfers in and out of the network per hour.
(2) Traffic Distribution: prediction of the number of transfers between the source and destination.
(3) Data Transmission Protocol: prediction of percentage by each transfer method.
(4) Routing Protocol: prediction of path-specific transmission count according to transfer method.

**Figure 4.**
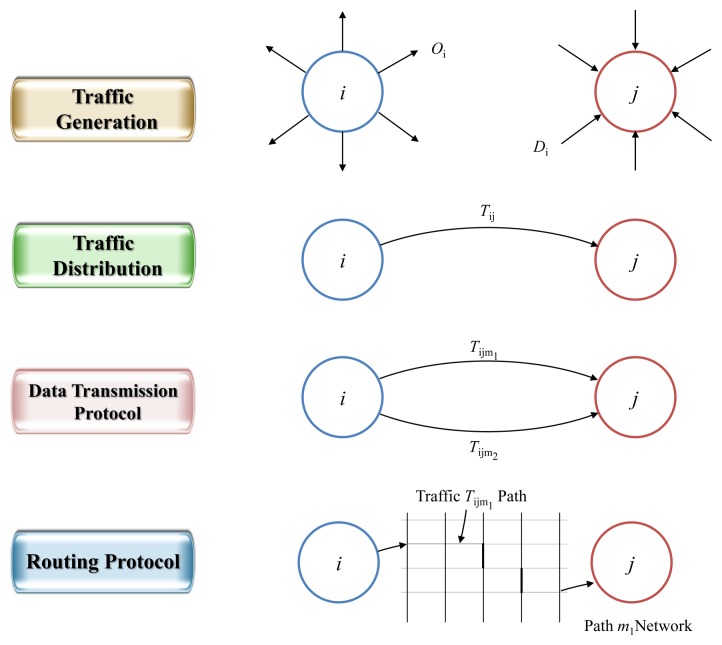
4 Steps of traffic demands analysis.

The above four elements could be predicted in sequential order when using Step 4 transportation demand analysis techniques. Using this technique, the estimate of the output results becomes the input for the estimation of the following steps. These steps are shown in Figure 4.

In this paper, we predict traffic generation and traffic distribution; for the rest of the data transmission/routing protocol it was assumed that any step can be used and that network demand was predicted in steps 1 and 2.

##### Traffic Generation

Cross-Classification Analysis, also known as Category Analysis, is a way to predict future network traffic by analyzing the incidence of average network traffic, the results of which will be different depending on various characteristics related to the occurrence of network traffic. The total traffic production of some areas in cross-classification analysis are calculated as the sum of the per-node traffic generation, and can be expressed as in Equation (6) below:

(6) $$O_{i}=\sum_{h}C_{h}R_{h}$$

- *O_i_* = The total traffic generation of area *i*
- *C_h_* = The number of nodes belonging to the category *h* of the characteristics that are classified
- *R_h_* = The average traffic generation of nodes belonging to the category *h*

##### Traffic Distribution

**Figure 5.**
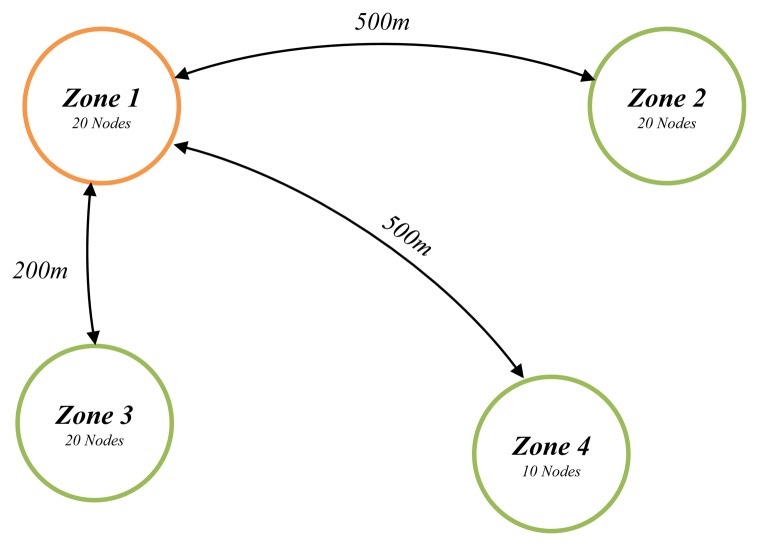
Gravity model example.

Network traffic between the C section and other sections is estimated using the gravity model. As shown in Figure 5, when comparing the network traffic between zones 1 and 2 with that between zones 1 and 4, the network traffic between zones 1 and 2 is greater than expected. This is because, although the distance between zone 1 and 2 and that between zone 1 and 4 are the same at 500 m, the number of nodes in zone 2 is 20, which is double the 10 nodes of zone 4. In addition, the number of nodes located within zone 1 and 2 is the same as that within zones 1 and 3, but the 500 m distance between zones 1 and 2 is more than twice as distant than the 200 m between zones 1 and 3, so it can be expected that the network traffic from zone 1 to zone 3 is higher than the network traffic from zone 1 to zone 4. This is due to the impacts of scale and distance, and the gravity model reflects this, which allows for traffic prediction.

The basic equation considering scale and distance impacts can be expressed as in Equation (7) below:

(7) $$T_{ij}=\frac{N_{i}N_{j}}{d_{ij}}$$

- *N_i_* = Node number in zone *i*
- *N_j_* = Node number in zone *j*
- *d_ij_* = The distance between zones *i* and *j*

The basic equation is limited as it is considers the characteristics of a WSN, for which traffic comes into the sink from the source. In other words, it places constraints for zone-specific total arrival traffic (*D_i_*). It must be transformed to satisfy the following constraint: the sum of the traffic (Σ*_i_T_ij_*) starting from all other zones arriving in zone *j* (sink) should be the same as the total amount of arrival traffic in zone *j* (*D_j_*). This is shown in the following Equation (8):

(8) $$\sum_{i}T_{ij}=D_{j}$$

The constraint is expressed in the following Equation (9):

(9) $$T_{ij}=O_{i}B_{j}D_{j}{d^{\beta }}_{ij}$$

- *B_j_* = Adjustment factor of destination zone *j*.

The adjustment factor *B_j_* may have different values for the destination zone in the (9). Using Equations (8) and (9), the adjustment factor *B_j_* can be derived as in the following Equation (10). Substituting Equation (11) into Equation (10), Equation (11) can be expressed as follows:

(10) $$B_{j}=\frac{T_{ij}}{O_{i}D_{j}{d^{\beta }}_{ij}}=\frac{\sum_{i}T_{ij}}{D_{j}\sum_{i}O_{i}{d^{\beta }}_{ij}}=\frac{D_{j}}{D_{j}\sum_{i}O_{i}{d^{\beta }}_{ij}}=\frac{1}{\sum_{i}O_{i}{d^{\beta }}_{ij}}$$

(11) $$T_{ij}={\text{D}}_{j}\frac{O_{i}{d^{\beta }}_{ij}}{\sum_{i}O_{i}{d^{\beta }}_{ij}}$$

Thus, in order to obtain the traffic volume *T_ij_*, the adjustment factor *B_j_* was first obtained by using Equation (10) and then, the traffic volume was calculated using Equation (10) or (11).

### 3.5. Data Transmission Time

The time *g_i_* taken to pass through zone *i* can be defined with a BPR function. Since the BPR function is easy to manipulate and is not asymptotic to any particular value, it has the advantage that the travel cost can be calculated for any traffic volume. In this paper, data modeling may be performed by applying the BPR function to the congestion of the network.

The BPR function developed by the United States Bureau of Public Roads shows how the traversal time changes depending on the ratio of traffic volume to the road capacity (Equation (12)):

(12) $$g_{i}=g_{i}(1+\alpha {\left(\frac{D_{i}}{C_{i}}\right)}^{\beta }$$

- *t* = transmission time
- *t*_0_ = transmission time for free path
- *D_i_* = traffic generation per area
- *C_i_* = network capacity (bps)
- α, β =parameters

*D_i_* is traffic generation per area and *C_i_* is network capacity. Thus, *g_i_* means transmission time.

**Figure 6.**
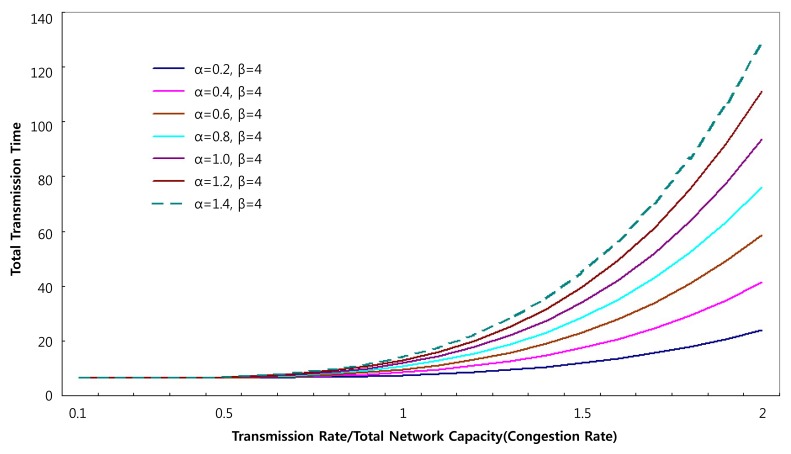
Transmission Time Changes by parameter α [40].

The BPR function has the advantage of fast-paced calculated results depending on the value of parameters α and β as shown in Figure 6. This may cause excessive calculation. So, it is important to determine the precise values of parameters α and β for the particular network types.

#### 3.5.1. Optimal Parameters Estimation: the Interval Reduction Method

Finding the optimal parameter values minimizes the difference between the estimated traffic from the model and the actual network traffic. We simulated several conditions for the WSNs as a training set. We consider the training set as a “miniaturized actual WSNs”. Nodes deployed random form; other conditions are default in NS-2. Conditions are shown in Table 3.

It can be expressed as a minimization problem as follows:

(13) $$\min Z\left(\alpha ,\beta \right)=\frac{1}{2}\sum_{b}{\left(T_{b}-\bar{T_{b}}\right)}^{2},\;v_{b}=v_{b}\left(t_{a}\left(\alpha ,\beta \right)\right)$$

The following Equation (14) can be obtained by substituting the above Equation (13) into the BPR Equation:

(14) $$\min Z\left(\alpha ,\beta \right)=\frac{1}{2}\sum_{b}{\left[\bar{V_{a}}-C_{a}{\left(\frac{\frac{T_{a}}{T_{a0}}-1}{a}\right)}^{-\frac{1}{\beta }}\right]}^{2}$$

**Figure 7.**
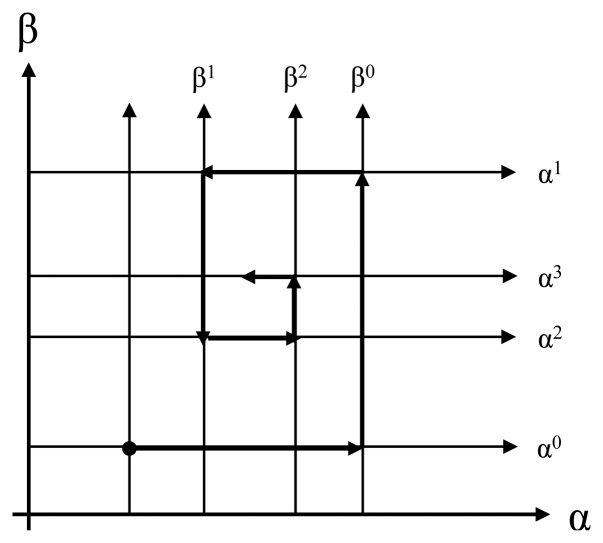
Parameter optimization.

The minimization problem can be solved through various previously-presented optimization techniques. In this paper, the interval expected to have an optimal solution is precisely analyzed by reduction of the iterative calculation, known as the golden section search. As shown in Figure 7, the initial parameter α^0^ was first fixed on the α-axis. The optimal β^0^ value was calculated by a golden section search on the β-axis. Then, the optimal α^1^ value was calculated again with β^0^ fixed. If this process is repeated several times, the optimal parameter α* and β* can be obtained. The process to obtain the overall optimal solution is detailed as follows:

[Step 1] Initialization: The actual amount of network traffic ((*V_a_*), initial (α^0^, β^0^) setting, *n* = 0
[Step 2] By fixing α*^n^*, β*^n^* can be calculated by using a golden section search.

The objective function used here is Equation (14) and *ν_b_* is the predicted network traffic.

[Step 3] By fixing β*^n^*, α*^n^*^+1^ can be calculated using a golden section search.
[Step 4] Convergence Review: if | α*^n^*^+1-^ − α*^n^*| < ε ‖|β*n*+1−β*^n^*| then stop.

If not, proceed to [Step 1].

#### 3.5.2. Network Type Classification

The values of α and β are expected to vary by the network type. Thus, the optimal α and β values can be found as in Section 3.5.1 after dividing the networks by speed and number of channels, as shown in Table 4.

## 4. Performance Evaluations and Results Analysis

### 4.1. Simulation Environment

Experiments were conducted by dividing the prediction of network traffic and transmission time into four sections: packet products per node, transmission generation by zones, estimation of parameters for rush hour prediction, and congestion prediction.

**Figure 8.**
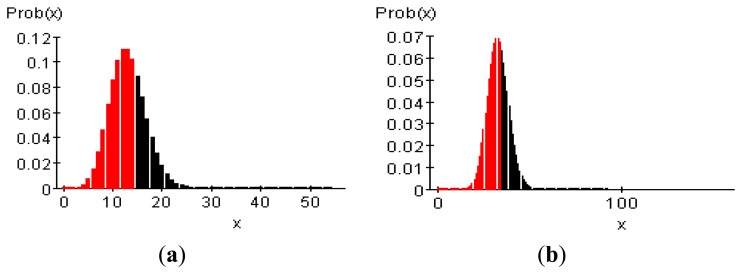
Poisson distribution for random event: (a) Event 10%∼15%; and (b) Event 30%∼35%.

Instead of building an actual network, the prediction model was tested by implementing a WSN using the IEEE 802.15.4 ns-2 package. The distance of one zone is 100 m, the total number of zones is 11, and the transmission radius of the nodes is set to 20 m. Between 10 and 20 nodes were placed randomly. When the node energy is 100%, the basic node energy is assumed to 1 J. It is assumed to be possible to operate for 100 min at 100%, and the nodes were randomly divided into three equal energy groups: 100%, 70%, and 40%. The node transmission time was set to be once per 10 s, and the event occurrence frequency was set to 30%∼35%, 10%∼15%, and 0% based, on 100 min. Events were randomly distributed with a Poisson model as shown in Figure 8. It was calculated under the same conditions for the proposed prediction model.

### 4.2. Results and Analysis

#### 4.2.1. Generated Packet

**Figure 9.**
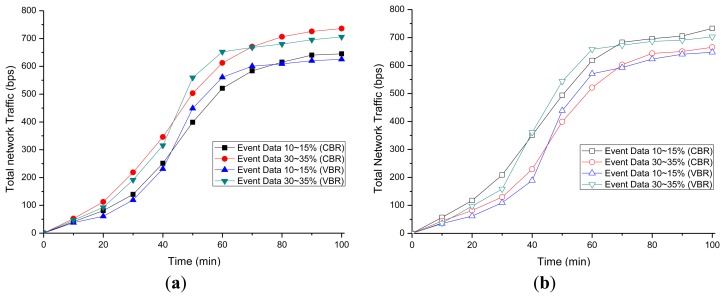
Total network traffic comparison—Nodes: (a) Congestion prediction model—total network traffic (b) NS-2—total network traffic.

The total packet production predicted in Section 3.4 was analyzed. The average number of packets produced by each node is shown in Figure 9. We use Constant Bit Rate (CBR) and Variable Bit Rate (VBR) to compare types of traffic. The prediction model is considered to be validated since the error rate of the total packet simulation product (b) was approximately 5% on average using the total number of packets (a) derived from the proposed prediction technique and NS-2.

**Figure 10.**
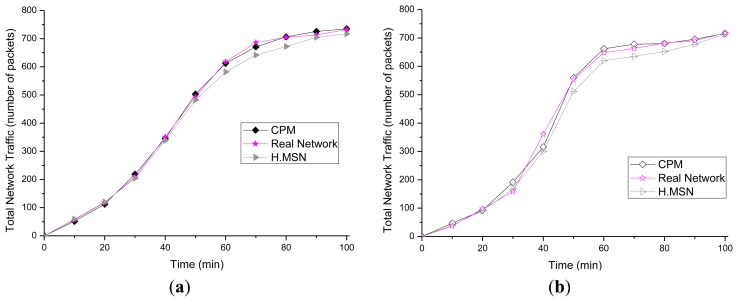
Traffic comparison (a) Traffic comparison in Constant Bit Rate (CBR); and (b) Traffic comparison in Variable Bit Rate (VBR).

The proposed traffic prediction model is compared with the real networks modeled in the ns-2 and traffic adaptive routing protocol for mobile sensor networks (H.MSN) [41]. The average number of packets produced by each node is shown in Figure 10. The proposed prediction model shows an average 3% difference with the real network in CBR simulation, and a 5% difference to that in VBR simulation. The H.MSN model shows an average 8% difference with the real-network in CBR, and a 12% difference to that in VBR simulation.

#### 4.2.2. Zone Traffic

**Figure 11.**
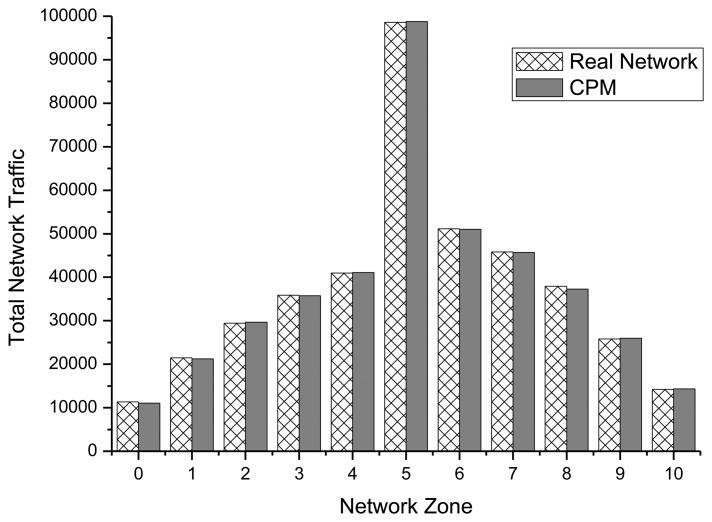
Total network traffic—zone.

The zone-specific total transmission predicted in Section 3.5 was also tested as shown in Figure 11. The total traffic that occurred in a zone over 100 min was represented as a graph of the derivative of the average value over 100 experiments. The error rate of the proposed prediction technique and the ns-2 model was shown to be approximately 7%.

#### 4.2.3. Bureau of Public Roads (BPR) Parameter Estimation

The results of the error rate for the transfer time prediction are shown in Table 5 by network category. The error rate was tested by comparing the network implemented in ns-2 to that of the prediction method proposed in this study. The error ratio was about 60% ± 30% (appropriate level), which does not represent a significant difference from the actual network implemented in ns-2.

**Figure 12.**
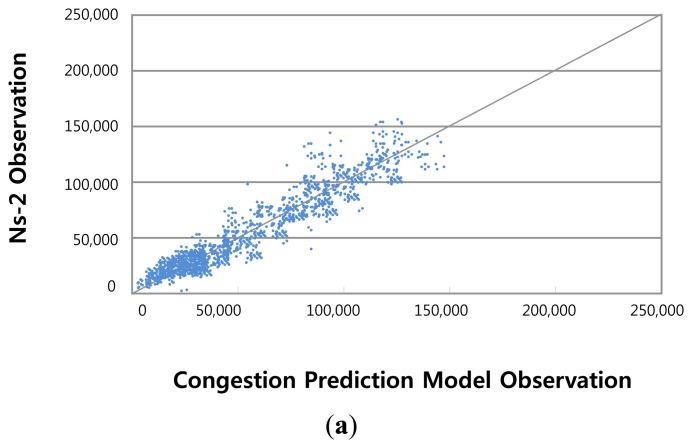

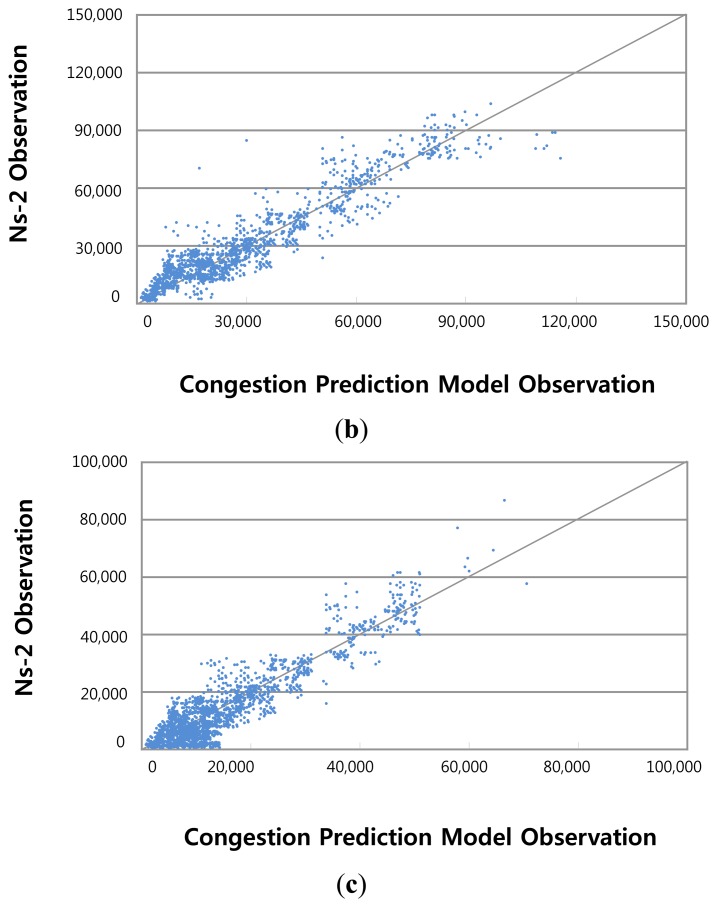
Comparisons between observed transmission times in the ns-2 and proposed models (a) comparison of high speed network transmission time observation; (b) comparison of medium speed network transmission time observation; and (c) comparison of low speed network transmission time observation.

Most of the observational data and the actual data converge in the middle, as shown in Figure 12. As can be seen in the figure, it reflects reality well.

#### 4.2.4. Data Transmission Time

**Figure 13.**
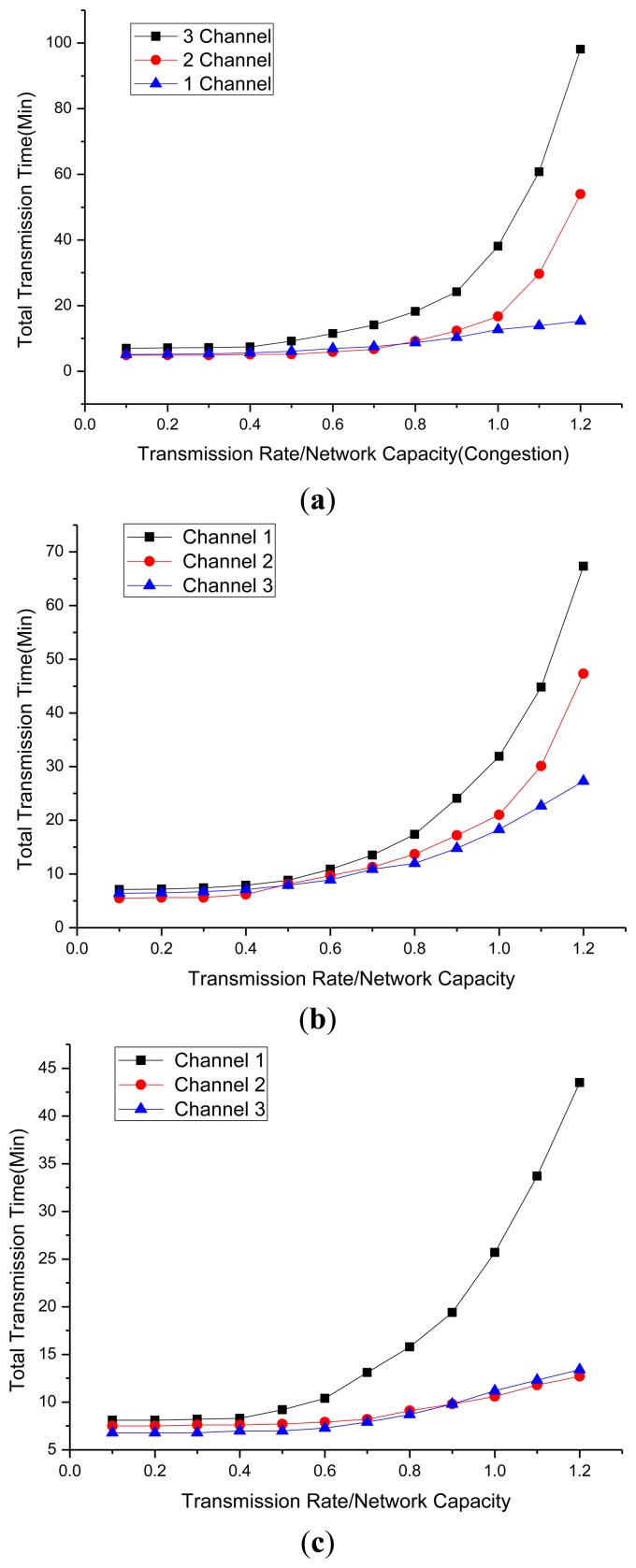
Congestion and transmission time comparison: (a) High speed network data transmission time; (b) Middle speed network data transmission time; and (c) Low speed network data transmission time.

Previous Simulation shows the similarity between real environment and prediction model. In this simulation, we analyzed transmission time regarding network capacity using prediction model. Total transfer time was tested by throughput as compared to network capacity using the BPR function presented in Section 3.5 and shown in Figure 13. α- and β- values calculated in Section 3.5.1 were used and a test was conducted depending on the network speed and the number of channels. The below Figure 13a–c show throughput compared to network capacity when the number of channels is more than one, two, and three in high-, medium-, and low-speed networks, respectively. High speed network is assumed to 100% speed when middle speed network is assumed to 70% speed and low speed network is assumed to 45% speed of high speed network.

In the high speed network, 2 channel network shows average 11.95% faster transmission time than 1 channel network, 3 channel network shows average 4.74% and 16.69% faster transmission time than 2 channel network and 1 channel network. In middle speed network, 2 channel network shows average 7.96% faster transmission time than 1 channel network, 3 channel network shows average 0.25% and 8.21% faster transmission time than 2 channel network and 1 channel network.

In the low speed network, 2 channel network shows average 5.58% faster transmission time than 1 channel network, 3 channel network shows average 2.65% and 8.23% faster transmission time than 2 channel network and 1 channel network.

This study shows that the estimated congestion shows a sensitive response to changes. It will therefore be able to predict congestion and can be used in the congestion zone in the future.

## 5. Conclusions

This paper presented a method for network modeling, packet production, and traffic prediction. There were several limitations to this study: (1) Network congestion costs are considered additional costs incurred during congestion according to the transmission time. It should have been estimated based on the actual transmission time, but WSN types differ greatly depending on application. Thus, the BPR function was used rather than specifying certain conditions; this use can lead to error; (2) The optimal value of the BPR function parameters depend on the characteristics of the network and the values presented in the national transportation data base, but it is important to find the optimal parameters for the network of interest because the overall trend can be changed by new elements; (3) The data used in this study were estimated and were not actual data and therefore may be inaccurate. These issues could have a significant impact on the estimated congestion prediction. However, improving the accuracy of the prediction through optimization with the simulation results of an actual network could address those problems. Thus, further improvements of the model should be made in future studies, since it predicts a trend rather than their own value. Additional studies will be performed on the basis of this study as follows:

(1) Node utility modeling should be performed and the effectiveness of the entire network should be analyzed accordingly.
(2) Congestion zones should be determined according to the analyzed effectiveness.
(3) The change in network utility should be analyzed depending on the design of the congestion zone.
(4) Lastly, changes in the transmission patterns of actual WSN network data should be identified and analyzed.

Many previous studies have focused on methodological aspects, congestion detection, and avoidance techniques [42,43]. The congestion pricing zone technique has the advantage of analyzing networks quantitatively through mathematical modeling, but it has not been studied extensively. In addition, this technique has previously been used in studies focused on short-term impacts such as congestion detection rather than congestion prediction. Therefore, various modeling techniques to efficiently control network congestion are presented here, but further theoretical research of various perspectives is needed. Theoretical studies of these long-term perspectives will determine how they can be used effectively in network design and maintenance.

## Figures and Tables

**Table 1.** Wireless Sensor Network (WSN) congestion control methods. ESRT: Event-to-Sink Reliable Transport; CODA: Congestion Detection and Avoidance; ACT: Adaptive Compression-based congestion control Technique; CCF: congestion control and fairness; SenTCP: A hop-by-hop congestion control protocol for wireless sensor networks; PCCP: priority-based congestion control protocol.

**Clear**	**Method**
Data flow control	Fusion [14], ESRT [15]
Congestion detection by using Queue status	CODA [16], ACT [17]
Packet service time	CCF [18]
Congestion information forwarding	SenTCP [19]
Node priority	PCCP [20]

**Table 2.** Traffic Analysis and Modeling method. AR: Auto-Regressive model; ARMA: Auto-Regressive Moving Average model; S-ARMA: Swam intelligence Auto Regressive Moving Average model.

**Types of Network**	**Traffic Prediction Method**	
Traditional	Markov, Poisson, Linear Regression, Time Series Forecasting	
WSN	Defend Attack	Intrusion Detection: [23], AR [24], ARMA [25], S-ARMA [21], Anomaly Detection: [26]
	Poison	Ma, Y. [27]
	CBR	Messier, G.G. [28]
	Markov	Shay, L.A. [29]
	Traffic Analysis [26]	Traffic Arrival Process Sequence Relations Data Traffic Load Distribution
	Others	Mobility-Aware [22]

**Table 3.** Training set condition.

**Classification**	**Option**
Number of nodes in 1 zone	100, 500, 1,000
1 Zone extent	10 m^2^, 100 m^2^, 500 m^2^
Periodic Data(number of times/s)	1/1, 1/5, 1/10, 1/60, 1/300
Event Data Probability (1 times per 1 min)	20%, 40%, 60%, 80%, 100%
Number of Sink in 1 zone	1, 5, 10
Bit Error Rate	20%, 40%, 60%, 80%, 100%

**Table 4.** Optimal *α*, β in network types.

**Network Speed**	**Number of Channel**	**Optimal α, β**
High	=1	α = 3.931, β = 5.316
	=2	α = 1.459, β = 1.943
	≥3	α = 3.210, β = 5.936
Middle	=1	α = 0.152, β = 4.020
	=2	α = 0.136, β = 3.984
	≥3	α = 0.581, β = 2.450
Low	=1	α = 1.896, β = 3.894
	=2	α = 0.430, β = 3.566

**Table 5.** Error rate for transfer time prediction by network category.

**Error (%)**		**High Speed Network**		**Medium Speed Network**		**Low Speed Network**	
		Observed value	Error Rate (%)	Observed value	Error Rate (%)	Observed value	Error Rate (%)
**Overestimation**	100+	18	2.7	64	4.6	109	4.0
	60∼100	44	6.5	72	5.2	252	9.2
	30∼60	76	11.3	103	7.4	322	11.8
	10∼30	115	17.1	279	20.2	320	11.7
	0∼10	98	14.5	227	16.4	439	16.0
**Underestimation**	−10 ∼ 0	92	13.6	201	14.5	549	20.1
	−30 ∼ −10	166	24.6	236	17.1	391	14.3
	−60 ∼ −30	55	8.2	117	8.5	158	5.8
	−60 ∼ −100	10	1.5	84	6.1	194	7.1
Total		674	100	1,383	100	2,734	100
